# Statistical models for evaluating suspected artefacts in long-term environmental monitoring data

**DOI:** 10.1007/s10661-018-6900-3

**Published:** 2018-08-29

**Authors:** Claudia von Brömssen, Jens Fölster, Martyn Futter, Kerstin McEwan

**Affiliations:** 1 Department of Energy and Technology, Division of applied statistics and mathematics, Swedish University of Agricultural Sciences, Uppsala, Sweden; 2 Department of Aquatic Sciences and Assessment, Section for Geochemistry and Hydrology, Swedish University of Agricultural Sciences, Uppsala, Sweden

**Keywords:** Level shift, Trend, Generalised additive mixed models, Method change

## Abstract

Long-term water quality monitoring is of high value for environmental management as well as for research. Artificial level shifts in time series due to method improvements, flaws in laboratory practices or changes in laboratory are a common limitation for analysis, which, however, are often ignored. Statistical estimation of such artefacts is complicated by the simultaneous existence of trends, seasonal variation and effects of other influencing factors, such as weather conditions. Here, we investigate the performance of generalised additive mixed models (GAMM) to simultaneously identify one or more artefacts associated with artificial level shifts, longitudinal effects related to temporal trends and seasonal variation, as well as to model the serial correlation structure of the data. In the same model, it is possible to estimate separate residual variances for different periods so as to identify if artefacts not only influence the mean level but also the dispersion of a series. Even with an appropriate statistical methodology, it is difficult to quantify artificial level shifts and make appropriate adjustments to the time series. The underlying temporal structure of the series is especially important. As long as there is no prominent underlying trend in the series, the shift estimates are rather stable and show less variation. If an artificial shift occurs during a slower downward or upward tendency, it is difficult to separate these two effects and shift estimates can be both biased and have large variation. In the case of a change in method or laboratory, we show that conducting the analyses with both methods in parallel strongly improves estimates of artefact effects on the time series, even if certain problems remain. Due to the difficulties of estimating artificial level shifts, posterior adjustment is problematic and can lead to time series that no longer can be used for trend analysis or other analysis based on the longitudinal structure of the series. Before carrying out a change in analytic method or laboratory, it should be considered if this is absolutely necessary. If changes cannot be avoided, the analysis of the two methods considered, or the two laboratories contracted, should be run in parallel for a considerable period of time so as to enable a good assessment of changes introduced to the data series.

## Introduction

Long-term water quality monitoring is of high value for environmental management as well as for research (Lindenmayer and Likens 2010; Fölster et al. 2014). Continuity in sampling procedures and analytical methods are crucial for the usability of monitoring data, when the usual challenge in evaluating a time series is to separate weak anthropogenic trends from large natural variation (Stålnacke and Grimvall 2001; Monteith et al. 2007; Erlandsson et al. 2008). Artefacts in data associated with changes in or problems with sampling or laboratory practice can lead to erroneous conclusions, as shown by Grimvall et al. (2014). Sometimes, however, discontinuities in time series cannot be avoided. Sampling locations might have to be moved for practical reasons, old analytical methods are replaced by new ones and there might be accidental flaws in laboratory practises. Furthermore, monitoring programmes are often conducted by different sub-contractors, leading to changes between laboratories. When evaluating time series of monitoring data, a credible method for testing whether known artefacts lead to significant changes in mean, variance or trend are needed, both to avoid reporting erroneous trends, but also to avoid rejecting real patterns.

Correct identification of level shifts or other discontinuities in time series is essential since their presence is easily confounded with trends or other effects (Beard et al. 1999). This is sometimes done by screening time series (e.g. Guzman et al. 2014). The most commonly used statistical method for such screening is the non-parametric Pettitt test (Pettitt 1979), which makes no a priori assumptions about the time point of level shifts. Cumulative sum (CUSUM) techniques (McGilchrist and Woodyer 1975) are also used, but traditionally, such techniques are recommended in adaptive real-time monitoring of individual systems with the goal of quickly detecting when levels deviate from their normal status (Mac Nally and Hart 1997) and not for retrospective screening. If the time point of a potential shift is known, *t* tests, ANOVA or the Kruskal-Wallis tests are sometimes used. A common limitation of all abovementioned methods is that they are constructed for situations where levels in the series are constant both before and after the level shift. The near ubiquitous presence of trends and seasonal variation implies that this assumption is hardly ever met in environmental time series.

Generalised additive models (GAMs, Hastie and Tibshirani 1986; Wood 2006) are applied to model trends in long time series, if the trend structure cannot be assumed to be linear. Since they give a more reasonable estimate of the temporal development in the series, they are also better equipped to estimate level shifts in such series (Bates et al. 2012; Ambrosino and Chandler 2013). A further advantage of GAMs is that effects of additional influencing factors can be accounted for using appropriate covariates, e.g. weather conditions during sampling. Replacing old chemical methods with newer ones that have lower analytical errors or improvements to laboratory practice will generally lead to a decrease in the variance of the time series, which can be accounted for in GAMs by including separate variance estimates for the different parts of the series. Temporal dependence in the series can be modelled by defining an autoregressive structure on the error term. When additive models include specific structures on the error term, the model class is called generalised additive mixed models (GAMMs).

When old methods are replaced by new ones, laboratories usually run the two methods in parallel in order to make a genuine assessment of any changes that are imposed on the resultant data series. The longer the overlap, the better are the possibilities to account for any unwanted level shifts due to the change in method.

In this study, we explore the use of GAMM models for detecting level shifts at known occasions in time series by simulation modelling. Further, we conduct a simulation study to evaluate how the presence of overlapping periods of observations improve and simplify the estimation of level shifts and which problems remain even after that. Finally, we use examples from the Swedish surface water quality monitoring programmes to illustrate data analysis in the presence of method or laboratory change.

## Statistical methods

In both the analysis of simulated and empirical data, generalised additive mixed models (GAMM, Hastie and Tibshirani 1986; Wood 2006) were used, since they can incorporate all important elements in the analysis, such as trends (long-term temporal variation), seasonality, the effects of short-term (e.g. meteorological) variation and level shifts at pre-specified time points, as well as temporal dependence between observations. GAMM also do not demand an a priori specification of the shape of the trend curve or the seasonal variation, as traditional linear models do. In this article, we use the model:

$$ {y}_i=\mu +{f}_1\left({\mathrm{time}}_i\right)+{f}_{2,\mathrm{cycl}}\left({\mathrm{month}}_i\right)+{\beta}_1\cdot {I}_{T_2i}+{\varepsilon}_i\kern1.5em i=1,\dots, n $$

The trend component *f*_1_(*time*_*i*_) is a smooth function over time modelled as a thin plate spline and seasonal variation *f*_2, cycl_(month_*i*_) is modelled using a cyclic cubic regression spline with an annual period. The level shifts were estimated as parametric changes at a known time point or a known time interval using an indicator (or dummy) variable ${I}_{T_2i}$ for the time point after the level shift, i.e. assuming the value 1 for all time points after and 0 before the level shift. If the two series overlap during a time period, the indicator variable represents one of the series and the level shift is assumed to be constant during the overlapping time period. If several potential level shifts are in the series, additional indicator variables can be included in the model. No information about meteorological or other potential forcing conditions was available here, but if necessary, this information can be included in the same way as above using spline or parametric functions.

The variance of the error term is allowed to vary for different parts of the series. Therefore, the variance-covariance matrix for the error term is modelled as:

$$ \Sigma =\left[\begin{array}{cc}{\sigma}_1^2{\mathrm{I}}_{T_1}& 0\\ {}0& {\sigma}_2^2{\mathrm{I}}_{T_2}\end{array}\right], $$

where ${\mathrm{I}}_{T_1}$ and ${\mathrm{I}}_{T_2}$ are indicator variables for time period 1 (before level shift) and 2 (after level shift) and ${\sigma}_1^2$ and ${\sigma}_2^2$ are the respective variances for these periods. Again, if there are several level shifts in the series, the matrix can be extended to include additional variance components.

In the empirical data analysis, the error term in the model is assumed to be normally distributed. To achieve normality, water chemistry variables are log-transformed if needed; therefore, any observations with a value of 0 were replaced by 1/10th of the smallest observation of the series. For the log-transformed series, the estimates of the level shift are to be interpreted as multiplicative rather than additive on the original scale. Observations that lay below the detection limit were replaced by half this limit. Serial correlation in the error term is estimated using an autoregressive process with lag 1 (AR(1)). The serial correlation was assumed to be the same before and after the potential level shifts. Residual variance was allowed to be different for different time periods.

The GAMM analyses were performed using the mgcv package in R (Wood 2006). Different residual variances are estimated by defining the according variance-covariance matrix using weights = varIdent (form = ~ 1|timeperiod), where timeperiod is an indicator variable as described above. Autocorrelation is estimated in the model using correlation = corAR1(form = ~ 1|timeperiod).

## Simulation study

A simulation study was conducted to determine the effect of a number of data set properties on the possibility to correctly estimate level shifts. The temporal structure and the approximate residual variance for the simulated data were extracted from one of the data sets described later (total nitrogen, Lule älv).

In the simulation study, we examined the following questions:

- How are the results affected by the location of the shift in the series with respect to an underlying trend?
- How does the correlation between the two methods in the overlapping time period affect the variance of the estimated level shift?
- How does residual variation influence the quality of the level shift estimate?
- How does serial correlation present in the error term affect the variation of the level shift estimation?

**Fig. 1:**
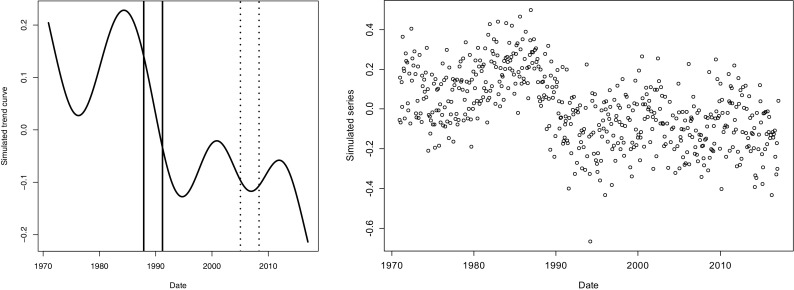
Left: Illustration of the underlying time structure used in simulations. The placement of the overlapping time periods is indicated by full lines for the ‘trend’ and with dotted lines for the ‘stable’ scenario (left). Right: A realisation of the ‘trend’ scenario

To illustrate the effect of non-stationarity in the time series on the estimate of a level shift, we simulated data from series with an underlying temporal structure. The simulated method change was placed at different location in this series illustrated (Fig. 1). The ‘trend’ scenario placed the potential level shift in a downward period, while the ‘stable’ scenario used a part of the series that varied less and lacked a monotonic temporal trend. Simulations of white noise random data were also made and denoted as ‘completely flat’. The location for the theoretical level shift in the ‘completely flat’ scenario was the same as for the ‘stable’ scenario. For testing the impact of overlapping time series, the maximum overlapping time period for the two methods was 40 observations and the simulated level shift is 0. Simulations were made to verify that the choice of magnitude of the level shift in the simulation was not important. Added level shifts lead to the same results as presented except for the magnitude of the estimated levels, i.e. the systematic error and variance of level shift estimate was not affected. Results of these simulations are not shown.

In the first set of simulations, we compared the average magnitude and variation of estimated level shifts when series were not overlapping versus when there was an overlapping period of up to 40 observations. The two series that overlap can, naturally, be correlated to different degrees with each other, depending on how similar the results of the new and the old method are. In this simulation study, we used correlations between series of 0.5 and 0.9.

In the simulation study, we let data be normally distributed and independent in time, i.e. we did not simulate serial correlations and the applied model did not contain any estimation of the serial correlation of the error term. An additional study was performed to show the effects of serial correlation in which the serial correlation coefficient was set to 0.6. Only the ‘completely flat’ scenario was simulated with serial correlation and correlation between series of 0.9. The applied model uses an AR(1) structure for the error term.

For all conducted simulations, 1000 series were generated. Residual variance was held constant for the simulated series and the estimated level shift was noted.

### Results of the simulation study

**Fig. 2:**
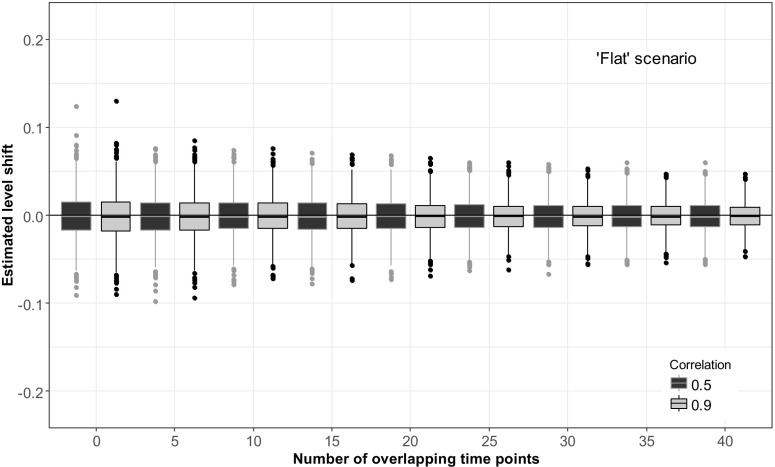
Estimated level shifts in a completely flat time series, i.e. a white noise series, plotted against the number of overlapping time points. The magnitude of the simulated level shift was 0. Black boxplots show the distribution of the estimated level shift if the correlation between the two methods is 0.5, and grey boxplots if the correlation is 0.9

In the reference scenario, the ‘flat’ scenario with no trend added to the simulation, estimated level shifts were stable (Fig. 2). The presence of 40 overlapping observations reduced the variance of the estimated level shift by about 40% compared to the variance when no overlap is present when the correlation between series is 0.9 and by about 20% when series have a correlation of 0.5.

**Fig. 3:**
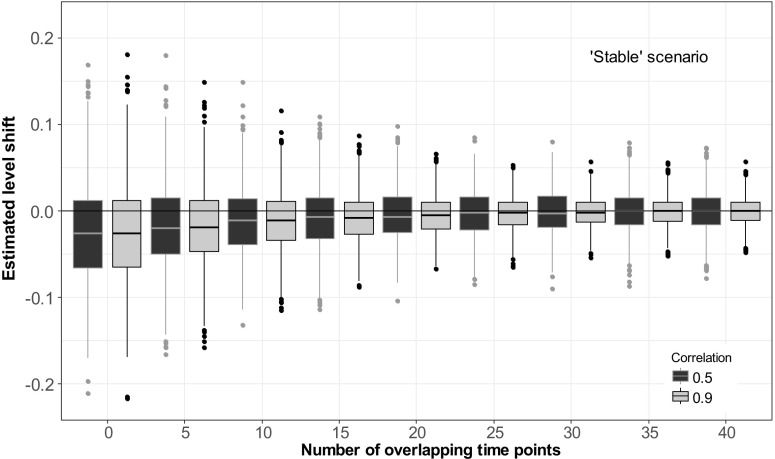
Estimated level shifts in a time series with the overlapping time period placed in a rather stable period plotted against the number of overlapping time points. The magnitude of the simulated level shift was 0. Black boxplots show the distribution of the estimated level shift if the correlation between the two methods is 0.5, and grey boxplots if the correlation is 0.9

**Fig. 4:**
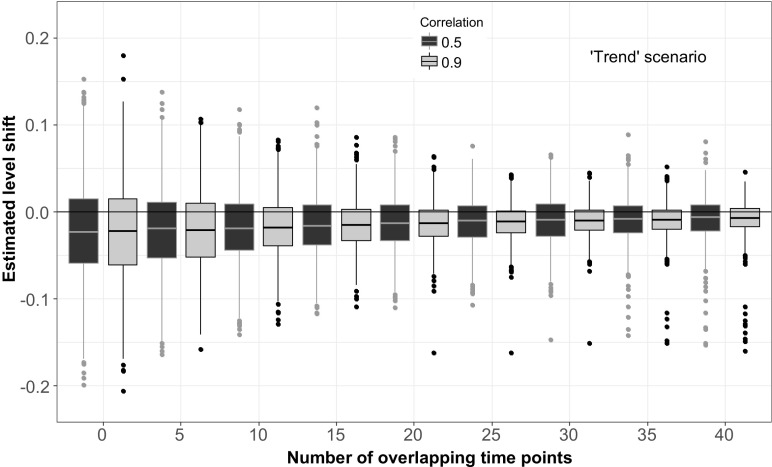
Estimated shifts in a time series with the overlapping time period placed at a downward period plotted against the number of overlapping time points. The magnitude of the simulated level shift was 0. Black boxplots show the distribution of the estimated level shift if the correlation between the two methods is 0.5, and grey boxplots if the correlation is 0.9

When series additionally exhibited a trend or other kind of slow variation, the existence of overlapping observations also had an effect on the estimated level. For series where the simulated shift occurred in a period of strong downward trend (Fig. 4), no overlapping observations led not only to high variation in the estimates but also to a bias. Both variation and bias in the estimates decreased when the number of overlapping time points increased. The mean estimated level shift when no overlap was present was − 0.023. For 40 overlapping observations, the mean shift was estimated to be − 0.0077. Further, the variance of the estimated level shift was reduced with more than 90% when an overlap of 40 observations was used, compared to no overlap (when the correlation between series was 0.9). Seventy-two out of 1000 simulations with no overlap lead to confidence intervals for the level shifts that did not cover 0. When the overlap was 40 observations, only 10 confidence intervals did not cover 0. The ‘stable’ scenario revealed similar results, but the systematic error was smaller and more quickly reduced as the number of overlapping observations increased (Fig. 3).

Generally, the estimate of the potential level shift is improved when the number of overlapping observations is increased. In the 1000 simulations, 859 give a level shift estimate closer to 0 when 40 observations are overlapping compared to no overlap at all. For the remaining 141 simulations, the difference between estimates in the two different situations is small except for 8 cases, which are clearly seen in Fig. 4. These estimations arise when the trend line is estimated to be linear instead of being close to the simulated temporal structure.

**Fig. 5:**
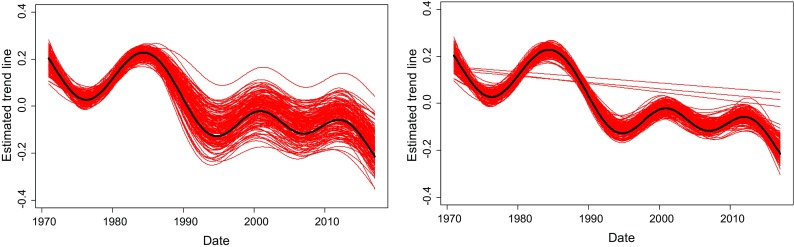
One hundred and fifty estimated trend curves as estimated for the trend scenario. No overlapping observations (left) and 40 overlapping observations (right). In some simulations, the trends were erroneously identified to be linear. The original trend line is printed in black

One hundred and fifty estimated trend curves are shown in Fig. 5. These trend curves were also influenced by the estimated level shift. For the situations with long overlap, the trend curves were more consistent with the true trend line, while the highly varying estimate of the level shift, naturally, led to a high variation also in the level of the trend curve. In the original simulated series without random error, a decrease of 7.3% was observed when comparing the mean of the series’ last 12 values with the mean of the first 12 values. If the estimated level shift is used to adjust or homogenise the series, this decrease can be over- or underestimated with up to four percentage points for the series without overlap. For the series with an overlap of 40 observations, the decrease can be underestimated with up to three percentage points in the situation where a linear trend was identified. Generally, the estimated level shift is much closer to the true value 0, which leads to an over- or underestimation of the estimated change in time of at most one percentage point.

**Fig. 6:**
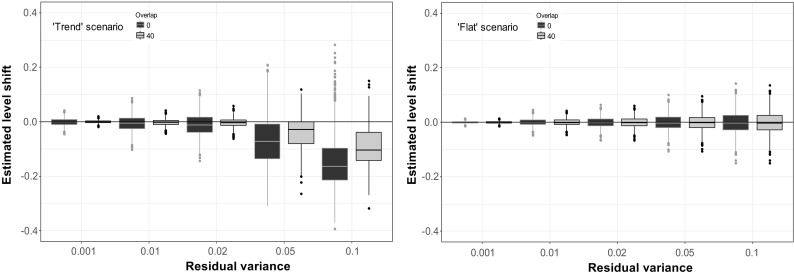
Estimated shifts in a time series with potential level shift placed at a downward period plotted (left) and in a completely flat series (right). No overlapping observations (black) or 40 overlapping observations (grey) are simulated

How well a level shift can be estimated, especially in a series that is not completely flat, is also strongly dependent on the residual variance. In Fig. 6, simulations were made for the ‘trend’ scenario using different magnitudes of residual variance. Simulations with no overlap and with an overlap of 40 observations are shown. Residual variance varies from 0.0001 to 0.1, while the simulated trend curve, as before, is changing with about 0.4 units. Simulations for the ‘flat’ scenario are given as comparison. As residual variation increased, the estimation of the level shift became more variable and biased.

Additionally, the trend curve is often no longer correctly identified, for example a linear trend was identified in 129 cases when no overlap was present and in 344 cases with overlap, when the residual variance was 0.1. The number of identified linear trends was dependent on the exact location of the level shift and the overlapping time period and not on the presence of absence of an overlap (results not shown). In the most extreme simulated case with residual variance 0.1 and the suspected level change set in the trend scenario, more than half of the confidence intervals for the level shift did not cover 0 for both the no-overlap situation (603 out of 1000) and the situation with 40 observations overlapping (511 out of 1000).

**Fig. 7:**
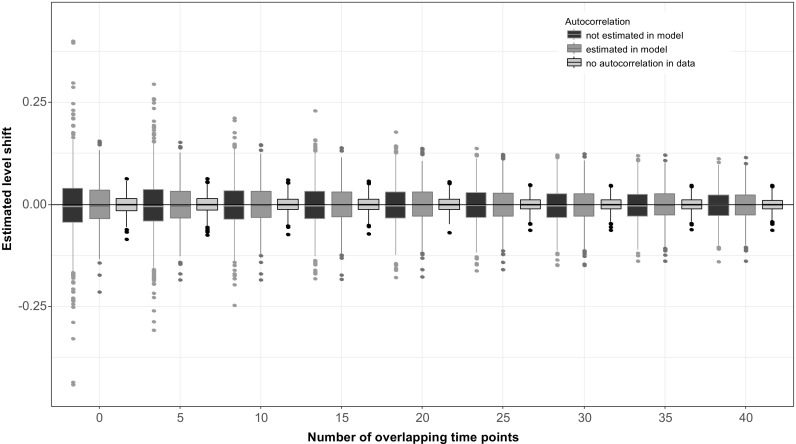
Estimated level shifts in a completely flat time series plotted against the number of overlapping time points. The magnitude of the simulated level shift was 0. One thousand simulations were made. Black boxplots show the distribution of the estimated level shift if the series contains serial correlation (*ρ* = 0.6), which is not accounted for in the analysis, dark grey boxplots show estimated level shifts if the serial correlation is accounted for in the model and light grey boxplot are used for reference for series with no serial correlation present

A separate simulation study was made using time series with relatively strong serial correlation (*ρ* = 0.6). When serial correlations of this magnitude were not estimated in the model, i.e. the observations are wrongly assumed to be independent, level shift estimates were highly variable (Fig. 7). Modelling and accounting for the serial correlation in the model reduced this variability, even though variation in the estimated levels shifts was still much stronger than in the reference series with no serial correlation. The effect of not modelling the serial correlation on the level shift was reduced when series overlapped for a substantial time period, but obviously the standard error of the level shift estimate would still be erroneous.

## Empirical data analysis

Swedish national monitoring of fresh water quality has been performed by the geochemical laboratory at the Swedish University of Agricultural Sciences since 1965 (Fölster et al. 2014). Using the same laboratory for most water quality analyses for more than 50 years has led to a level of high continuity and good documentation of historical flaws and method changes. For a few subprogrammes (e.g. monitoring of the Swedish great lakes), the monitoring went out for bidding, which led to changes in laboratory over time for some of the series. All chemical analyses followed standard methods.

The data sets used in this paper illustrate two different scenarios, where the estimation of a potential level shift is important:

- Some overlap: the chemical analysis method is replaced by a new one. Separate methods are run in parallel during a limited time period.
- No overlap: different laboratories perform the chemical analysis, with no field samples analysed in common.

To illustrate scenario a, time series of total nitrogen were used. In Sweden and elsewhere, total nitrogen was historically assessed by adding the sum of nitrite and nitrate nitrogen (ISO 15923-1:2013) to Kjeldahl nitrogen (Jönsson 1966). The latter includes the fractions organic nitrogen and ammonium nitrogen. The sum of Kjeldahl nitrogen, nitrate and nitrite is here denoted as TN-Kj. In 2007, this method was replaced by the total nitrogen bound (TNb) method (SS-EN 12260:2004). In the TNb method, all nitrogen fractions included in the older method are combusted into nitrous oxide that are detected by chemiluminescence. During the first years of using the TNb method, some adjustments were made. In July 2008, the calibration curve was adjusted to improve precision for low nitrogen values. In September 2009, handling of samples with higher amounts of particulate matters was improved, but only in March 2010, the use of a magnetic stirrer guaranteed that particulate matter in the samples was handled efficiently. The adjustments can be noticed in the data by the presence of some very low observations prior to September 2009.

Time series from three streams with different levels of total nitrogen (TN) were included in the study. Lule älv in the North of Sweden has low TN levels (around 200 μg/l) with monthly observations available between December 1970 and January 2017. Dalälven is situated in mid-Sweden and has slightly higher TN levels (300–400 μg/l) due to higher levels of natural organic nitrogen. Monthly observations for Dalälven were available for July 1965 to February 2017. Domneån is situated in an agricultural area in Southern Sweden and has high TN values (between 1000 and 1500 μg/l) with organic nitrogen as the main fraction (between 400 and 1200 μg/l) but often with high levels of nitrate and nitrite (0–1000 μg/l). Monthly observations from Domneån were available from November 1969 to February 2017. For all three streams, the methodology for measuring TN was replaced in January 2007. The overlapping time period for the old and new methods was 40 months for Dalälven and Lule älv, but only 12 months for Domneån. No values below the current detection limit were noted for these series.

For scenario b, data from four streams running into the lake Vättern were used (Forsvikån/Forsvik, Mjölnaån/Vättern, Röttleån/Röttle, Motalaström/Motala). These rivers were monitored between 1980 and 2014 and during this time, the chemical analyses were conducted by several laboratories. Laboratory 1 conducted the analysis until March 2004. Laboratory 2 conducted the analysis between April 2004 and December 2009. A new laboratory was contracted for analysis from January 2010 to March 2010; however, as there were only three observations obtained from this laboratory, these observations were removed from this study. From April 2010 onwards, laboratory 4 conducted the analysis. Observations are made monthly for each of the series. A small number of values below the detection limit were observed for Forsvikån (5) and Röttleån (2). For Motalaström, 18 observations were below the detection limit and 17 of these were observed after April 2010. All contracted laboratories were accredited by SWEDAC and used similar analytical methods.

Even though methods and processes at different laboratories were comparable, there are a number of different reasons why levels or variation in time series from different conductors could vary. Differences can occur in the calibration process, where e.g. the instruments are optimised for low or high concentrations, respectively. Other reasons for deviating measurements from different laboratories include the practice of shaking the sample bottle before taking out a subsample for analysis or stirring of the sample during the analysis. This can be particularly important for analysis of e.g. total phosphorus and total organic carbon in samples with a high content of particulate matter. When measuring pH of samples high in carbon dioxide, the degree of aeration that affects the results may vary between different laboratories. For reactive analytes such as ammonia and phosphate, the temperature and time between taking the sample and doing the analysis is important. Finally, the skills and devotion of the laboratory staff set the limit for the quality of the results.

### Results of empirical data analysis

#### Scenario a: some overlap: the chemical analysis method is replaced by a new one

When overlapping periods are available for both methods, the use of all available observations should be most beneficial in the statistical analysis. However, there can be reasons to abandon this principle, e.g. if the old method is obsolete and needs to be replaced as soon as possible or if the new method is expected to have some running-in problems and cannot be relied on initially. To illustrate the effect of these different approaches on the analysis of TN, we fit GAMM for these situations respectively: (i) all data were used, i.e. measurements for TN-Kj and TNb were used where available, leading to an overlapping period of between 12 (Domneån) and 40 months (Lule älv and Dalälvan), (ii) TN-Kj is replaced by TNb in January 2007 and (iii) TN-Kj is replaced by TNb in May 2010.

**Fig. 8:**
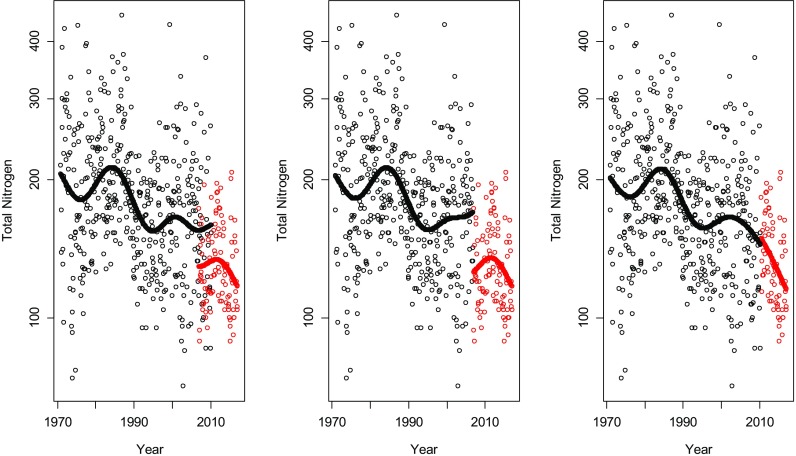
The series of total nitrogen in Lule älv. Black dots indicate measurements of TN-Kj and red dots measurements of TNb. In the plot to the left, all data is used including an overlapping period of 3 years and 4 months. In the middle plot, TN-Kj is replaced by TNb in January 2007 and in the right plot, the replacement is made in May 2010, i.e. in the end of the overlapping period

**Fig. 9:**
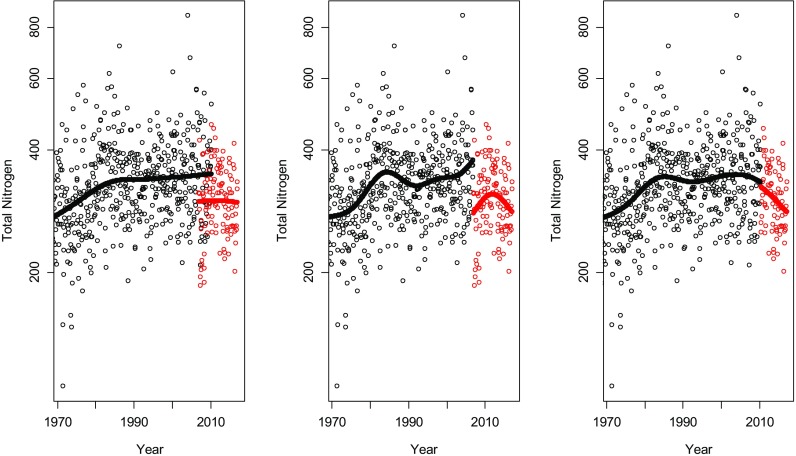
The series of total nitrogen in Dalälven. Black dots indicate measurements of TN-Kj and red dots measurements of TNb. In the plot to the left, all data is used including an overlapping period of 3 years and 4 month. In the middle plot, TN-Kj is replaced by TNb in January 2007 and in the right plot, the replacement is made in May 2010, i.e. in the end of the overlapping period

**Fig. 10:**
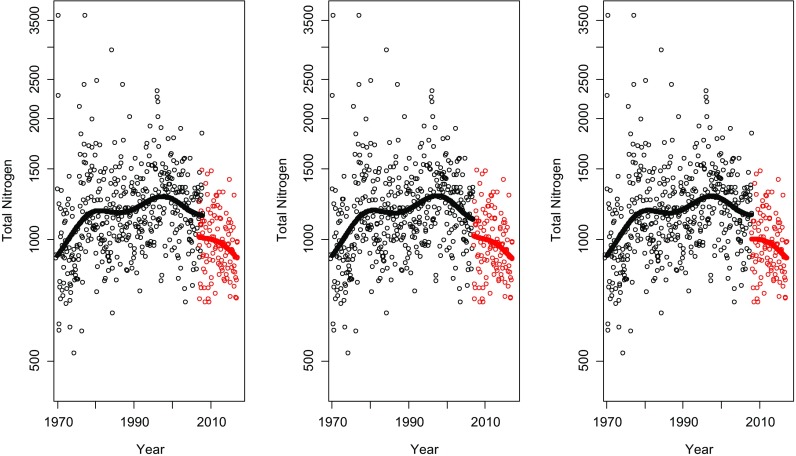
The series of total nitrogen in Domneån. Black dots indicate measurements of TN-Kj and red dots measurements of TNb. In the plot to the left, all data is used including an overlapping period of 1 year. In the middle plot, TN-Kj is replaced by TNb in January 2007 and in the right plot, the replacement is made in January 2008, i.e. in the end of the overlapping period

The fitted trend curve for each of the three rivers is shown in Figs. 8, 9 and 10. In Tables 1, 2 and 3, the estimations of the magnitude of the level shifts are given as well as their confidence intervals. Since TN was log-transformed prior to the analysis, the estimates give the multiplicative change and hence a relative difference of 1 means no level shift. Additionally, separate residual variances were estimated for TN-Kj and TNb. Estimated autocorrelation coefficients vary little for the three different approaches. The correlation between the TN-Kj and TNb series in the overlapping time period is also given.

**Table 1:** Estimation results for three models for Lule älv. The estimated difference is multiplicative giving the level of TNb in relation to the level of TN-Kj. Separate variances were estimated for the two methods

		All data used	TN-Kj before 2007, TNb after that	TN-Kj before May 2010, TNb after that
Estimated relative difference		0.83	0.74	1.04
95% confidence interval of level shift		0.74–0.94	0.61–0.90	0.85–1.3
Variance	TN-Kj	0.087	0.085	0.088
	TNb	0.049	0.048	0.045
Autocorrelation coef.		0.24	0.289	0.25
Correlation between methods in overlap		0	–	–

**Table 2:** Estimation results for three models for Dalälven. The estimated difference is multiplicative giving the level of TNb in relation to the level of TN-Kj. Separate variances were estimated for the two methods

		All data used	TN-Kj before 2007, TNb after that	TN-Kj before May 2010, TNb after that
Estimated relative difference		0.86	0.74	0.98
95% confidence interval of level shift		0.79–0.94	0.64–0.85	0.86–1.1
Variance	TN-Kj	0.048	0.047	0.047
	TNb	0.034	0.032	0.029
Autocorrelation coef.		0.257	0.239	0.23
Correlation between methods in overlap		0.5	–	–

**Table 3:** Estimation results for three models for Domneån. The estimated difference is multiplicative giving the level of TNb in relation to the level of TN-Kj. Separate variances were estimated for the two methods

		All data used	TN-Kj before 2007, TNb after that	TN-Kj before 2008, TNb after that
Estimated relative difference		0.88	0.91	0.87
95% confidence interval of level shift		0.78–0.99	0.80–1.05	0.76–1
Variance	TN-Kj	0.060	0.060	0.060
	TNb	0.042	0.041	0.041
Autocorrelation coef.		0.149	0.146	0.15
Correlation between methods in overlap		0.95	–	–

The three analysed time series are located at very different geographical locations with different influencing factors. Domeån is highly influenced by intense agricultural activities and snowmelt dominates the hydrological regimes of Dalälven and Lule älv. Nonetheless, all three series show similar effects during the time period where both methods were used. For the series, where TN-Kj was measured until April 2010, an analysis based on TN-Kj as long as possible indicated that there was basically no difference between the two methods (Figs. 8 and 9, Tables 1 and 2), while replacing TN-Kj with TNb in January 2007 or using all data indicates that TNb lies at levels of about 20–30% lower than TN-Kj. A decrease of this amount could be enough to fulfil the goals for N-load reduction set by national authorities. Also, the correlation between the methods during the overlapping period varied strongly and seemed to be dependent on nitrogen levels, starting with Domneån with highest nitrogen levels and a very strong correlation between methods, to Lule älv with low level that showed no correlation at all between TN-Kj and TNb (Tables 1, 2 and 3).

Separate variances for the two parts of the series, i.e. before and after the method change, were computed and it can be observed that variation for TNb is generally lower than that for TN-Kj amounting to a 30–40% decrease in residual variance (Tables 1, 2 and 3).

**Fig. 11:**
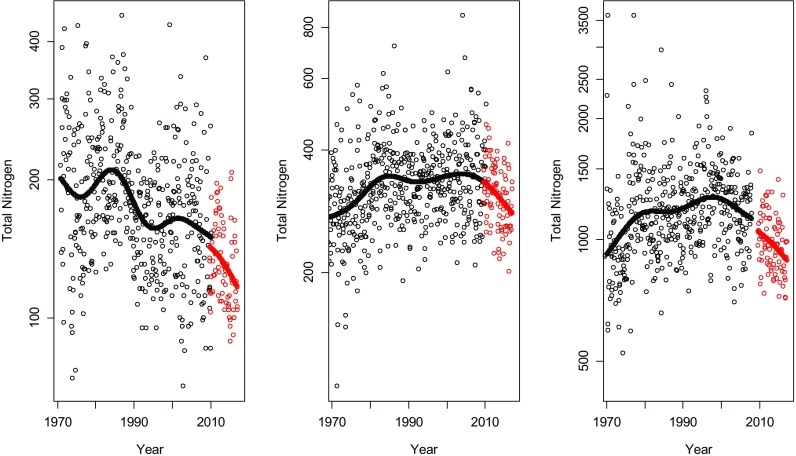
The estimated trend curves and level shifts if the observations with potential measurement errors for TNb are removed. Lule älv (left), Dalälven (middle) and Domeån (right)

When data prior to September 2009 was removed to deal with problems in the handling of particulate matter, model fits in Fig. 11 were obtained. The remaining overlapping time period for Dalälven and Lule älv was 8 months, while for Domneån, a gap of 20 month arose. In these models, TNb is estimated to about 95–100% of the level of TN-Kj and, thus, indicating consistent levels before and after the method change. No adjustment of the series using the estimated level shift is recommended in this case prior to a trend analysis or other statistical analysis that uses the longitudinal structure of the data. Removal of uncertain observations prior to September 2009 and the use of overlapping observations after that should lead to a reliable analysis.

#### Scenario b: no overlap: different laboratories perform the chemical analysis

In the present data set, no detailed information was available on how laboratories handled and analysed samples. The goal of the following analysis was, thus, to see if there were any obvious changes in level when a new laboratory was contracted. We analysed time series of ammonia and pH in the four river mouth stations.

**Fig. 12:**
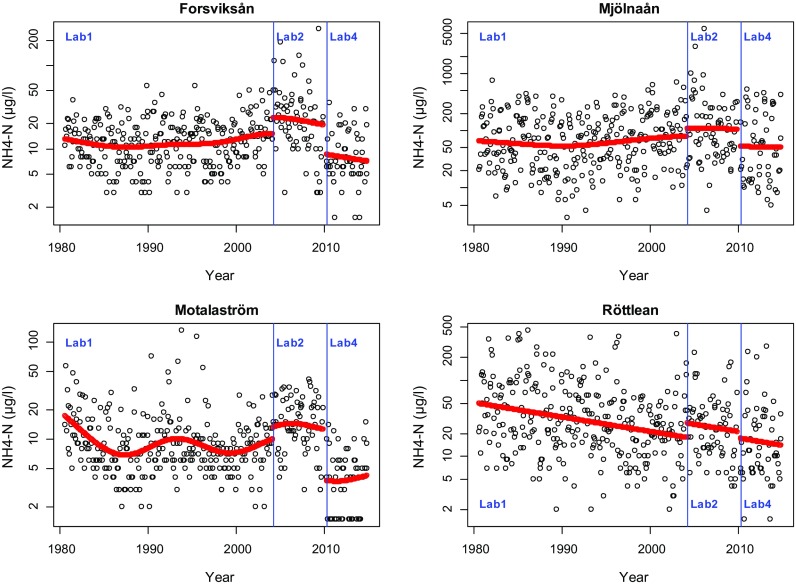
Ammonia in four rivers leading into Vättern. Estimated trend curves with level shifts when the conducting laboratory is changed. Observe that units on the *y*-axis are on the log-scale

When analysing ammonia, we saw a slight increase of level for laboratory 2 compared to laboratory 1, but this can easily be caused by climatic variations or by chance, since none of the changes were strongly significant (Fig. 12; Table 4). Comparing laboratory 4 with laboratory 2, a decrease in level was observed for Forsviksån and Motalaström, the two rivers with the lowest levels of ammonia. In these two rivers, the level of ammonia measured by laboratory 4 was only about 30–45% of the level measured by laboratory 2 at the time point of change in the beginning of 2010. It needs to be noted that there was a gap of 3 months in the time series at this point due to the removal of observations made by laboratory 3. For Motalaström, at the time after April 2010 (laboratory 4), 17 observations below the detection limit (3 μg/l) were noted and replaced with half this detection limit. The specific handling of these values influences the estimated level shift between laboratories 2 and 4.

**Table 4:** Estimated differences (multiplicative) in ammonia between laboratories and separate variance estimates for each laboratory. A relative difference of 1 means no level shift

		Forsviksån Forsvik	Mjölnaån Utl. Vättern	Motalaström Motala	Röttleån Röttle
Estimated relative difference	Lab 1 to Lab 2	1.54	1.36	1.32	1.54
	Lab 2 to Lab 4	0.45	0.50	0.30	0.82
95% confidence interval of level shift	Lab 1 to Lab 2	1.2–2.3	0.73–2.5	0.80–2.2	1.07–2.22
	Lab 2 to Lab 4	0.27–0.74	0.25–1	0.17–0.52	0.57–1.18
Variance	Lab 1	0.308	0.987	0.41	0.71
	Lab 2	0.93	1.35	0.43	0.72
	Lab 4	0.358	1.06	0.44	0.70
Autocorr. coef.		0.17	0.33	0.08	0.19

The estimated trend curve for all rivers except Röttleån looked similar, which was expected since they lie close to each other geographically. For Röttleån, a linear trend was identified. Röttleån has a higher residual variation then Forsviksån and Motalaån in relation to the series mean, but a lower variation than Motala ström.

**Fig. 13:**
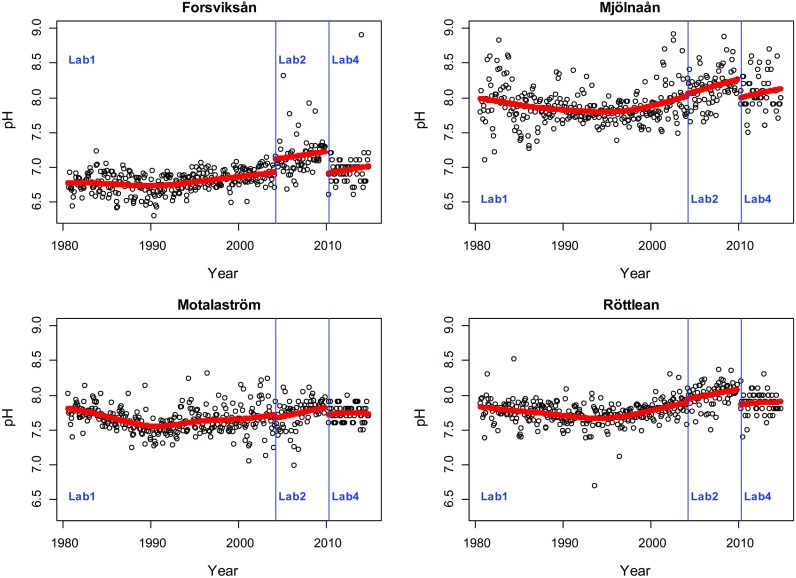
pH in four rivers leading into Vättern. Estimated trend curves with level shifts when the conducting laboratory is changed

The analysis for pH was done on untransformed data and therefore the estimated differences are interpreted as additive and given in Table 5. As for ammonia, any shift between laboratory 1 and laboratory 2 is negligible, but in several series, the estimates indicate a shift in level for pH after laboratory 4 was employed (Fig. 13). This change was significant in all series except Motalaström and had a magnitude of about 0.2–0.3 units. Again, a gap of 3 month was present in the series at this time point.

**Table 5:** Estimated differences (additive) in pH between laboratories and separate variance estimates for each laboratory. A difference of 0 means no level shift

		Forsviksån Forsvik	Mjölnaån Utl. Vättern	Motalaström Motala	Röttleån Röttle
Estimated difference	Lab 1 to Lab 2	0.19	0.03	− 0.051	0.049
	Lab 2 to Lab 4	− 0.32	− 0.27	− 0.11	− 0.22
95% confidence interval of level shift	Lab 1 to Lab 2	0.08–0.23	− 0.15–0.22	− 0.19–0.09	− 0.05–0.14
	Lab 2 to Lab 4	− 0.50–− 0.14	− 0.49–− 0.06	− 0.23–0.017	− 0.33–− 0.11
Variance	Lab 1	0.038	0.057	0.035	0.023
	Lab 2	0.082	0.063	0.047	0.0259
	Lab 4	0.09	0.061	0.020	0.025
Autocorr. coef.		0.29	0.33	0.27	0.03

## Discussion

Long-term environmental monitoring gives rise to time series that are influenced by many factors, including anthropogenic factors (e.g. changing pollution levels, long-term climate trends), natural factors (e.g. seasonal variation or weather conditions during or prior to sampling) and artificial factors (changes in sampling, chemical analysis or conductors). Statistical analysis of such series should include as many of these factors as possible to be able to identify the important drivers behind what is observed. If known that a series has undergone a substantial change in method, it is essential to allow the estimation of an artificial level shifts or change in residual variance at the corresponding time points to enable reliable trend analysis.

In this paper, we estimated the trend using generalised additive mixed models (GAMM, Hastie and Tibshirani 1986; Wood 2006), a robust approach which does not demand a pre-specification of the relationship between the response variable and time. It allows the addition of explanatory factors in the model, such as known break points or influences from weather conditions. With the GAMM model, it is also possible to estimate different residual variances for separate time periods before and after a break point and can, therefore, account for differences not only in the mean but also in the variation of the series. Similar models were used by Ambrosino and Chandler (2013), who observed that the level shift estimate is influenced by how well the trend curve is estimated and showed that too naïve estimates often lead to over- or underestimation of the artificial level shift. In the present study, we investigated how the presence of overlapping observations simplifies the estimation of an artificial level shift and how the quality of this estimation is influenced by the position of the level shift in a series with a trend and by present residual variation.

Our simulation study showed that estimates of level shifts improve as the overlapping period is extended. How reliable the shift estimates are is however dependent on a number of factors: (i) the presence and magnitude of a simultaneous long-term trend, e.g. caused by steady increasing or decreasing pollution levels, (ii) the correlation between the series in the overlapping time period, (iii) the presence of autocorrelation and, of course, (iv) the size of the residual variance. Especially when the residual variance is high and a trend is present, the estimates of the level shift can be both biased and have a high variation. Using such estimates to correct or homogenise series can have a negative effect on the analyses that follows this adjustment, as they can introduce erroneous conclusions both regarding the estimated magnitude of a trend and the attribution of temporal changes to the correct source.

How to choose the length of the overlapping time period cannot be generally addressed, but is dependent on which kind of change in methodology is made. In the presented case study, the consequences of fundamental change in laboratory methodology for measuring total nitrogen were evaluated and a long overlapping period was chosen. In other cases, when the change between methods is rather an adjustment, or well tested methods are used before and after the change, much shorter overlapping series will, in most cases, be sufficient.

Considering the start-up problems associated with the new method in our first case study, the overlap was necessary or even too short. In this case, the entire overlapping period could not be used for estimation of the level shift, but the first part was used to identify data quality problems and to determine when the change from one to the other method can safely be done in a trend analysis or other statistical assessment of the series. In our study, we could show that the method change did not lead to a significant change in level after data quality problems were removed. This allows further analysis of trends for these series.

A special situation is the change of laboratory that is conducting the chemical analysis. In Sweden, all laboratories used for national monitoring are accredited and use comparable methods. Thus, all laboratories should produce comparable results. However, there are many factors that can still affect the outcome, such as different ways of handling the sample, age and model of the analysing instrument or conscientiousness of the laboratory personnel. In the same way as when exchanging the method itself, it is essential to have overlapping time periods to account for systematic differences in level or in variation between the approaches. Since, however, the reason for the replacement of laboratory often is to cut analysis cost, overlapping is usually not chosen. In this paper, we investigate if a common analysis for all stations that have undergone the laboratory change can be used to identify potential shifts. We found that there were some indications of obtaining different levels from the analysis of different laboratories, especially for pH, but since no detailed information about laboratory practices, such as the degree of aeration, was available, we cannot draw any conclusions if the suspected differences were caused by such, by anthropogenic factors or by climatic variations. To facilitate comparison between laboratories, further investigations should be made in the exact way the chemical analyses are conducted in the different laboratories. Without such investigation, further analysis using the time series structure of the data should be conducted with care.

To evaluate the effects of method change and to make reliable trend estimates in series that are affected by such method changes, it is essential to employ flexible models that can fit the underlying trend curve as well as other factors that can influence the level and variation of the series. A decrease in variation at the same time point as a method change can be reasonable if the new method leads to improved chemical analysis. For most series for environmental pollution data, it is also important to be able to estimate serial correlation, since observations cannot be assumed to be independent. Similarly, as the estimates of level shifts, autocorrelation estimates are influenced by the present trend structure (e.g. Chandler and Scott 2011) and it is, therefore, essential to be able to model all these features in the same model, which can be obtained by the employed GAMM models. At least one of the series in our case study contained a substantial number of values below the detection limit during a limited time period and a statistically correct handling of these, instead of a mere replacement with half the detection limit, would improve the estimate of the level shifts. This can be done by incorporation censored data in GAMM (Stasinopoulos and Rigby 2007).

In our study, we assume that the time point of break in the series is known, which is reasonable when methods or laboratories are exchanged consciously. The tested models can also be applied when instrument malfunctions are detected but the start date of this is unknown. The series can then be screened with similar models testing different break point positions and different magnitudes of level shift with the goal to find the fit that best describes the data. Such an approach is described by Libiseller et al. (2005) using a non-parametric smoother fitted by penalised least squares and can be conducted with the models used in this paper as well. Other statistical methods that are sometimes used to identify the location of break points in series, such as Pettitt’s test or CUSUM methods, are built on the assumption of constant levels before and potentially after the break and are usually not adequate to use for environmental data that seldom meet this requirement.

Many long-term studies in the environmental sciences use the Mann-Kendall (MK) test for trend assessment (e.g. Futter et al. 2014; Huser et al. 2018). While the MK test is robust to many of the common problems with environmental time series including missing and non-normally distributed data (Hirsch and Slack 1984), it does not consider the effects of the types of level shifts presented here. Further work is needed to explore how the MK test and other widely used non-parametric trend estimators such as Sen’s slope respond when this type of artefacts are present.

## Conclusions

To be able to make statements about the development of water quality or other environmental series over time, it is essential to have internally consistent sets of observations (Beard et al. 1999; Lindenmayer and Likens 2010; Fölster et al. 2014). For a number of different reasons, this is not always possible and trend analysis should not be done thoughtlessly on series that have potential inconsistencies.

In this paper, we used GAMM to examine series for level shifts due to method change or change in laboratories that perform the chemical analyses. We applied generalised additive models that allow the estimation of a smooth trend and a parametric level shift, which, in theory, allows us to remove the level shift from the data and make trend tests or other analysis on seemingly consistent series. However, we also showed that this procedure can be problematic, especially if there is an upward or downward trend at the same time period as the potential shift and the residual variation is high. A genuine examination of the series, including all potential factors, such as anthropogenic, natural and artificial influences is necessary to make a balanced assessment of available data. In best case, we can identify a situation when changes do not lead to a detectable level shift and thereby the series can be approved for further analysis. If the magnitude of a level shift is to be estimated, the analysis should be based on observations made within an overlapping time period and, if the same shift is expected for several series, a common analysis for all of these series should be made.

If the goal of a change is to replace an old method with a new and better procedure, there is still a very good possibility to estimate and handle a potential level shift. Generally, both methods are run in parallel for some time to ensure a high-quality assessment of the change made. In other scenarios, such as malfunction of instruments or if new laboratories take over the analysis, it is, naturally, not possible or not chosen to run the analyses in parallel and therefore a thorough analysis of level shifts is not possible. There is no way to come round the problem that instruments sometimes malfunction and the access of a good quality assurance system with dense checks is essential. To willingly introduce potential level shifts should however be avoided. If economic reasons, for example, are behind the wish to change laboratories, the value of a time series with such shifts needs to be taken into account. There is a substantial risk of ending up with a series that cannot be used for a thorough and credible assessment of changes over time.

## References

[CR1] Ambrosino C, Chandler RE (2013). A nonparametric approach to the removal of documented inhomogeneities in climate time series. Journal of Applied Meteorology and Climatology.

[CR2] Bates BC, Chandler RE, Bowman AW (2012). Trend estimation and change point detection in individual climatic series using flexible regression methods. Journal of Geophysical Research-Atmospheres.

[CR3] Beard GR, Scott WA, Adamson JK (1999). The value of consistent methodology in long-term environmental monitoring. Environmental Monitoring and Assessment.

[CR4] Chandler RE, Scott EM (2011). Statistical methods for trend detection and analysis in the environmental sciences.

[CR5] Erlandsson M, Buffam I, Folster J, Laudon H, Temnerud J, Weyhenmeyer GA, Bishop K (2008). Thirty-five years of synchrony in the organic matter concentrations of Swedish rivers explained by variation in flow and sulphate. Global Change Biology.

[CR6] Fölster J, Johnson RK, Futter MN, Wilander A (2014). The Swedish monitoring of surface waters: 50 years of adaptive monitoring. AMBIO.

[CR7] Futter MN, Valinia S, Löfgren S, Köhler SJ, Fölster J (2014). Long-term trends in water chemistry of acid-sensitive Swedish lakes show slow recovery from historic acidification. AMBIO.

[CR8] Grimvall A, von Brömssen C, Lindstrom G (2014). Using process-based models to filter out natural variability in observed concentrations of nitrogen and phosphorus in river water. Environmental Monitoring and Assessment.

[CR9] Guzman JA, Chu ML, Starks PJ, Moriasi DN, Steiner JL, Fiebrich CA, McCombs AG (2014). Upper washita river experimental watersheds: data screening procedure for data quality assurance. Journal of Environmental Quality.

[CR10] Hastie T, Tibshirani R (1986). Generalized additive models. Statistical Science.

[CR11] Hirsch RM, Slack JR (1984). A nonparametric trend test for seasonal data with serial dependence. Water Resources Research.

[CR12] Huser BJ, Futter MN, Wang R, Fölster J (2018). Persistent and widespread long-term phosphorus declines in boreal lakes in Sweden. Science of the Total Environment.

[CR13] Jönsson E (1966). The determination of Kjeldahl nitrogen in natural water. Vattenhygien.

[CR14] Libiseller C, Grimvall A, Walden J, Saari H (2005). Meteorological normalisation and non-parametric smoothing for quality assessment and trend analysis of tropospheric ozone data. Environmental Monitoring and Assessment.

[CR15] Lindenmayer DB, Likens GE (2010). The science and application of ecological monitoring. Biological Conservation.

[CR16] Mac Nally R, Hart BT (1997). Use of CUSUM methods for water-quality monitoring in storages. Environmental Science & Technology.

[CR17] McGilchrist CA, Woodyer KD (1975). Note on a distribution-free CUSUM technique. Technometrics.

[CR18] Monteith DT, Stoddard JL, Evans CD, de Wit HA, Forsius M, Hogasen T, Wilander A, Skjelkvale BL, Jeffries DS, Vuorenmaa J, Keller B, Kopacek J, Vesely J (2007). Dissolved organic carbon trends resulting from changes in atmospheric deposition chemistry. Nature.

[CR19] Pettitt AN (1979). A non-parametric approach to the change-point problem. Journal of the Royal Statistical Society. Series C, Applied Statistics.

[CR20] Stålnacke P, Grimvall A (2001). Semiparametric approaches to flow normalization and source apportionment of substance transport in rivers. Environmetrics.

[CR21] Stasinopoulos, D. M., Rigby, R. A. (2007). Generalized additive models for location scale and shape (GAMLSS) in R. *Journal of Statistical Software 23*. 10.18637/jss.v023.i07.

[CR22] Wood S (2006). Generalized additive models an introduction with R.

